# Immunity of an Alternative Host Can Be Overcome by Higher Densities of Its Parasitoids *Palmistichus elaeisis* and *Trichospilus diatraeae*

**DOI:** 10.1371/journal.pone.0013231

**Published:** 2010-10-13

**Authors:** Gilberto Santos Andrade, José Eduardo Serrão, José Cola Zanuncio, Teresinha Vinha Zanuncio, Germano Leão Demolin Leite, Ricardo Antonio Polanczyk

**Affiliations:** 1 Departamento de Biologia Animal, BIOAGRO, Universidade Federal de Viçosa, Viçosa, Brazil; 2 Departamento de Biologia Geral, Universidade Federal de Viçosa, Viçosa, Brazil; 3 Instituto de Ciências Agrárias, Universidade Federal de Minas Gerais, Montes Claros, Brazil; 4 Departamento de Proteção de Plantas, Universidade Estadual Paulista Júlio de Mesquita Filho (UNESP), Universidade do Estado de São Paulo, São Paulo, Brazil; Institut de Pharmacologie et de Biologie Structurale, France

## Abstract

Interactions of the parasitoids *Palmistichus elaeisis* Delvare & LaSalle and *Trichospilus diatraeae* Cherian & Margabandhu (Hymenoptera: Eulophidae) with its alternative host *Anticarsia gemmatalis* (Hübner) (Lepidoptera: Noctuidae) affect the success or failure of the mass production of these parasitoids for use in integrated pest management programs. The aim of this study was to evaluate changes in the cellular defense and encapsulation ability of *A. gemmatalis* pupae against *P. elaeisis* or *T. diatraeae* in adult parasitoid densities of 1, 3, 5, 7, 9, 11 or 13 parasitoids/pupae. We evaluated the total quantity of circulating hemocytes and the encapsulation rate *versus* density. Increasing parasitoid density reduced the total number of hemocytes in the hemolymph and the encapsulation rate by parasitized pupae. Furthermore, densities of *P. elaeisis* above 5 parasitoids/pupae caused higher reduction in total hemocyte numbers. The encapsulation rate fell with increasing parasitoid density. However, parasitic invasion by both species induced generally similar responses. The reduction in defensive capacity of *A. gemmatalis* is related to the adjustment of the density of these parasitoids to their development in this host. Thus, the role of the density of *P. elaeisis* or *T. diatraeae* by pupa is induced suppression of cellular defense and encapsulation of the host, even without them possesses a co-evolutionary history. Furthermore, these findings can predict the success of *P. elaeisis* and *T. diatraeae* in the control of insect pests through the use of immunology as a tool for evaluation of natural enemies.

## Introduction

Parasitism, and the development of the parasitoid lifestyle, depends on the host being able to meet the nutritional requirements of the parasite and the ability of the parasite to overcome the immune response of the host [1], [2]. Moreover, genetic factors and the host's innate ability to respond to the parasite invasion determine the degree of the host's resistance to parasitism [1], [3]–[4]. Therefore, parasites rely on their ability to suppress the cellular and humoral defenses of their hosts [5].

Hemocytes are the main defense cells of insects and originate during embryonic development, while maintenance and differentiation of prohemocytes continue the production and circulation of these cells in the hemolymph in adult insects [2]. Membrane receptors in hemocytes recognize invaders and promote subsequent protective cellular reactions including phagocytosis, nodulation or encapsulation [6]. The most common types of insect hemocyte are prohemocytes, plasmatocytes, granulocytes and oenocytoids, although variants can be recognized in different insect species [7, 8–6].

Proteins and peptides also recognize pathogens and then adhere to, and alter, the molecular properties of the invader's cell membrane or cell wall [9]. They then produce proteolytic and toxic molecules that kill the invading parasitoids, bacteria or fungus. Melanin is the final product of these prophenoloxidase cascade reactions, leading to the death of the pathogens [10]. This melanogenesis generates nitrogen and oxygen reactive species that damage the structure of proteins and DNA, and are fatal to pathogens. If invading organisms are to live and develop successfully within a host they must overcome these host defenses [1], [11], [12], [13], [14].

The use of monofilament nylon or micro-injections of Sephadex beads are two methods for estimating the encapsulation of insects *in vivo* [15], because the capacity to encapsulate abiotic material is related to the ability to encapsulate by non-self recognition [16].The most appropriate method for bioassays in pupae has been the use of implants of nylon monofilament, because microinjections of liquid into pupa with Sephadex microspheres could damage the pupal beg [15].

Parasitoids such as Eulophidae (Hymenoptera) can be used to regulate populations of agricultural insect pests [17], [18]. Effective use of these natural enemies in integrated pest management programs relies on good basic knowledge of the parasitoid's biology [19], [20], because the mass production of parasitoids for release depends on our being able to produce them in quantity within suitable hosts [21].

*Palmistichus elaeisis* Delvare & LaSalle 1993 (Hymenoptera: Eulophidae) parasitize the pupae of Bombycidae, Noctuidae, Arctiidae and Tenebrionidae [22]. *Trichospilus diatraeae* Cherian & Margabandhu 1942 (Hymenoptera: Eulophidae) parasitize the pupae of Crambidae and Noctuidae [23], [24]. Both parasitoids have been studied with a view to using them to control pests of agricultural crops and forests [22], [24]–[28]. On the other hand, the reproductive success of these parasitoids has been dependent on varying numbers of them according to host species [22], [23], [24]. One possible explanation is that the lack of co-evolutionary relationship between alternative hosts and these parasitoids hinder the suppression of host immune defense, causing failures in the mass production or even possible failures in the use of these natural enemies to control insects pests.

Understanding the host immune response allows us to predict the success of individual parasitoid species in alternative hosts. The objective of this study was to evaluate the cellular defense of *Anticarsia gemmatalis* pupae (Hübner, 1818) (Lepidoptera: Noctuidae) and their ability to encapsulate invaders when exposed to different densities of the parasitoids *P. elaeisis* and *T. diatraeae*, so as to choose a suitable alternative host for mass rearing of these natural enemies.

## Materials and Methods

### Host

Eggs of *A. gemmatalis* were placed in 1100 mL plastic vials and after hatching the larvae were fed on an artificial diet [29] at 25±1°C, 70±10% relative humidity, and 14 h photophase. At the end of fifth instar, larvae were transferred to 1100 mL pots, 1/5 filled with sand previously sterilized at 150°C for two hours, to allow pupation. The pupae were transferred to wooden boxes (30×30×30 cm) supplied with nutrient solution (10.5 g of honey, 60 g of sucrose, 1.05 g of nipagin and 1.05 g of ascorbic acid diluted in 1.05 L of distilled water) embedded in a cotton ball for feeding of the hatched adults. Eggs were collected on white paper sheets placed inside the wooden boxes and transferred to vials provided with the artificial diet.

### Parasitoids

*Palmistichus elaeisis* and *T. diatraeae* were obtained from the Laboratory of Biological Control of Insects at the Universidade Federal de Viçosa, Minas Gerais and kept at 25±1°C, 70±10% relative humidity, and f 14 h photophase. Six females of *P. elaeisis*, 72 h after emergence and eight newly emerged *T. diatraeae* females were each presented with one pupa of *A. gemmatalis* for 24 h in glass tubes (14×2.2 cm) containing drops of honey for the parasitoids to feed on. The number and age of parasitoid females chosen were those with the best parasitism rates [24, unpublished data].

### Hemocyte count in hosts

Pupae of *A. gemmatalis* (229.72±5.12 mg and 24 h old) were exposed to 1, 3, 5, 7, 9, 11 or 13 adult parasitoids to test the effect of parasitoid densities on the host immune response. The pupae were exposed to 72 h old, mated females of *P. elaeisis* [26] or newly emerged mated *T. diatraeae* females. At these ages the parasitoids have mature eggs suitable for oviposition (data not shown). After this period, the pupae were rinsed with 1% sodium hypochlorite for five seconds and then with distilled water. Four microliters samples of pupal hemolymph were collected with micropipettes from a small incision in the thorax and transferred to 20 µL of buffer (98 mM NaOH, 186 mM NaCl, 17 mM Na_2_ EDTA and 41 mM citric acid, pH 4.5) to prevent the hemocytes aggregation [30].

The 4 µL haemolymph samples were stained with Giemsa and the total numbers of hemocytes, granulocytes, plasmatocytes and other hemocytes types were counted [10], [6]–[30] using a hemocytometer (Neubauer) with a 40 x objective lens.

### Encapsulation Rate

Nylon filaments (2×0.2 mm) were sterilized with 1% sodium hypochlorite, washed with distilled water and implanted into pupae of *A. gemmatalis* [29]. These pupae were individually placed in glass tubes (14.0×2.2 cm) and exposed to 1, 3, 5, 7, 9, 11 or 13 mated females of *P. elaeisis* or *T. diatraeae* for 24 h. Pupae not exposed to parasitoids were used as a control. The filament implants remained inserted into the pupae for 48 h and were then removed, mounted on slides and observed under a light microscope [15].

Images of the nylon implants were made with a photographic camera, Canon PowerShot™ A640, and processed using the computer program RemoteCapture Task ™ with the following settings: white balance (day light); exposure compensation (+1); flash exposure level (zero); metering mode (evaluative); ISO speed (auto); AE Mode (Program AE). The spectral signature of the implants was measured using ImageJ software,National Institutes of Health, USA [31]. The mean absorbance value was adopted as a measure of the rate of encapsulation with values from zero to 255. The mean absorbance of the samples was adjusted by subtracting it from 255, owing to the fact that the computer program indicates the highest encapsulation rate as zero and the lowest one as 255 [31], [32]. The arbitrary values of the implants were adjusted by discounting the background [31].

### Statistical analysis

The comparison between parasitoids were conducted using the nonparametric Wilcoxon test (p≤0.05). The hemocyte values at different densities of each parasitoid were compared using regression analysis with computer program SigmaPlot 10.0 (p≤0.05) with 15 repetitions. The dimensionless values for the rate of encapsulation were compared by regression analysis with 20 repetitions (p≤0.05).

## Results

**Figure 1:**
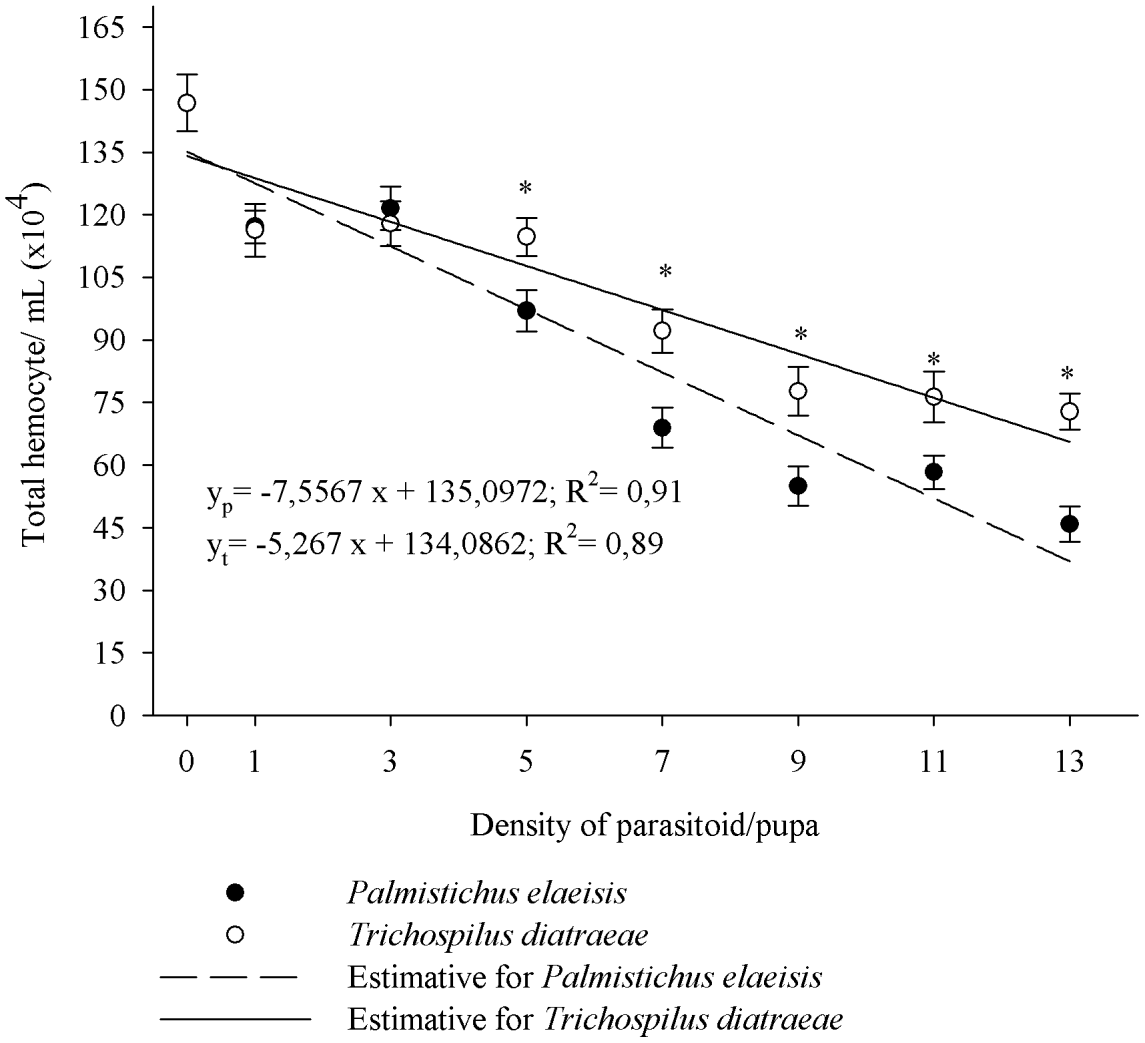
Hemocyte (mean ± se) of *Anticarsia gemmatalis* pupae parasitized by densities of *Palmistichus elaeisis* or *Trichospilus diatraeae*.

Increasing the density of adult *P. elaesis* reduced the total number of hemocytes in the hemolymph of *A. gemmatalis* pupae with 117.13×10^4^ and 45.85×10^4^ cells mL^−1^, at the lowest and highest densities, respectively (F = 68.6945, P≤0.01) (Figure 1). Parasitism by *T. diatraeae* also reduced the total number of hemocytes in the hemolymph of *A. gemmatalis* with 116.3×10^4^ cells mL^−1^ and 72.8×10^4^ cells mL^−1^ at lowest and highest parasitoid densities, respectively (F = 49.2905, P = 0.0004). Furthermore, the reduction in total circulating hemocytes was greater under parasitism by *P. elaeisis* in the densities of 5, 7, 9, 11 and 13 (p≤0.05) (Figure 1).

**Figure 2:**
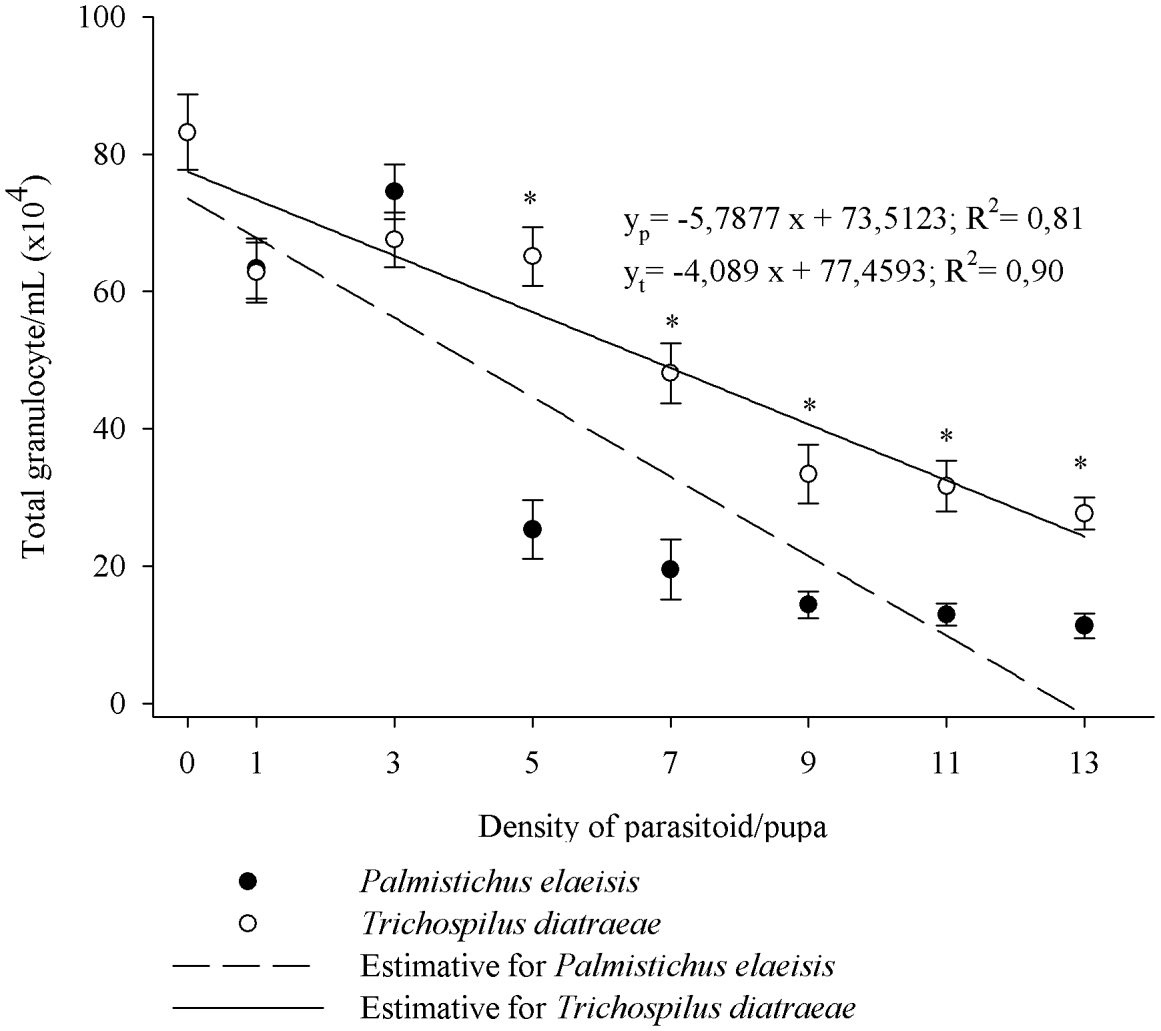
Total granulocyte (mean ± se) of *Anticarsia gemmatalis* pupae parasitized by densities of *Palmistichus elaeisis* or *Trichospilus diatraeae*.

The number of circulating granulocytes in *A. gemmatalis* pupae decreased linearly as a function of increasing density of *P. elaeisis*, from 63.35×10^4^ to 11.32×10^4^ cells mL^−1^ (F = 25.2121, P≤0.01) and similarly for *T. diatraeae*, from 62.77×10^4^ to 27.65×10^4^ cells mL^−1^ (F = 55.1502, P≤0.01) from the lowest to the highest densities of parasitoids, respectively (Figure 2). *Palmistichus elaeisis* showed a greater ability to reduce circulating granulocytes in the hemolymph of *A. gemmatalis* at densities higher than 5 parasitoids/pupae than *T. diatraeae* (p≤0.05).

**Figure 3:**
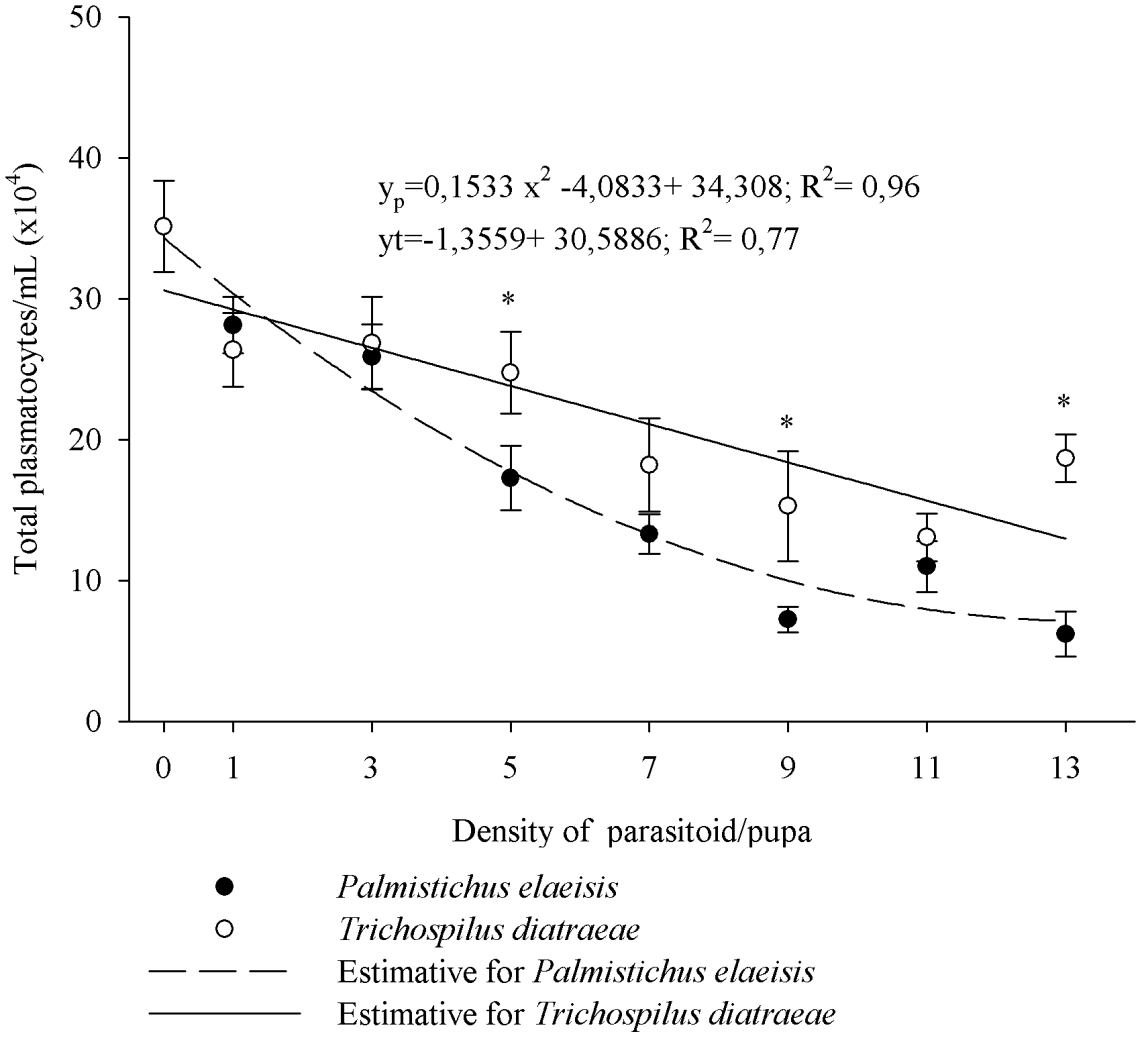
Total plasmatocytes (mean ± se) of *Anticarsia gemmatalis* pupaeparasitized by densities of *Palmistichus elaeisis* or *Trichospilus diatraeae*.

The number of circulating plasmatocytes was lower with increasing densities of *P. elaeisis* (F = 63.3011, P≤0.01) and *T. diatraeae* (F = 19.6201, P≤0.01) (Figure 3). *Palmistichus elaeisis* showed greater ability to reduce circulating plasmatocytes in the hemolymph of *A. gemmatalis* at densities of 5, 9 and 13 parasitoids/pupae and a similar reduction at densities below 5 females/pupae. Moreover, *T. diatraeae* showed a linear reduction in the number of circulating plasmatocytes in the hemolymph of *A. gemmatalis* with increasing parasite density (Figure 3).

**Figure 4:**
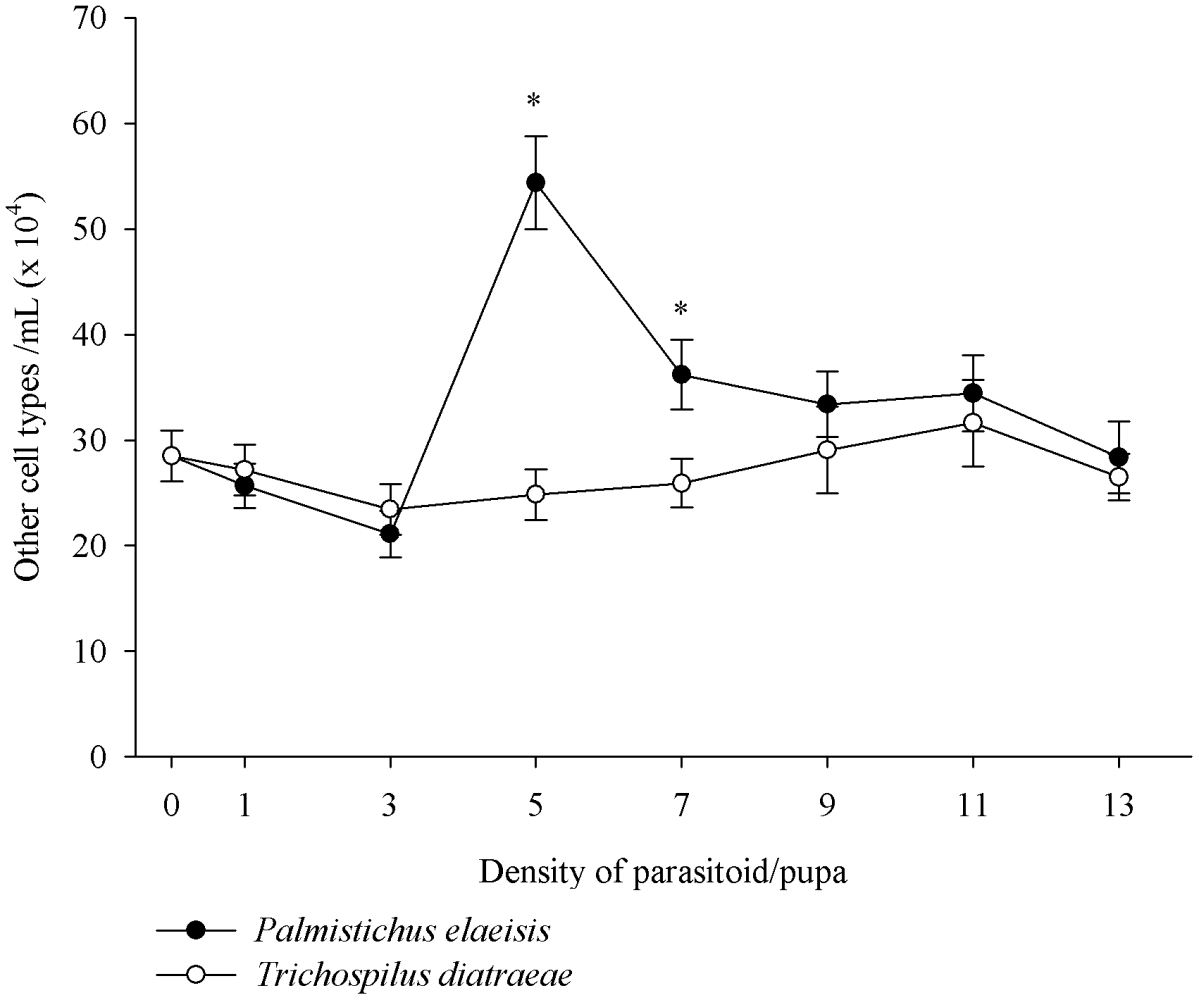
Total of other cell types (mean ± se) of *Anticarsia gemmatalis* pupae parasitized by densities of *Palmistichus elaeisis* or *Trichospilus diatraeae*.

There is an irregular pattern in the reduction of other types of circulating hemocytes in the hemolymph of *A. gemmatalis* with increases in parasitoid density (Figure 4). The number of these cells was higher when parasitized by *P. elaeisis* than *T. diatraeae* at densities of 5 and 7 parasitoids/pupae, respectively.

**Figure 5:**
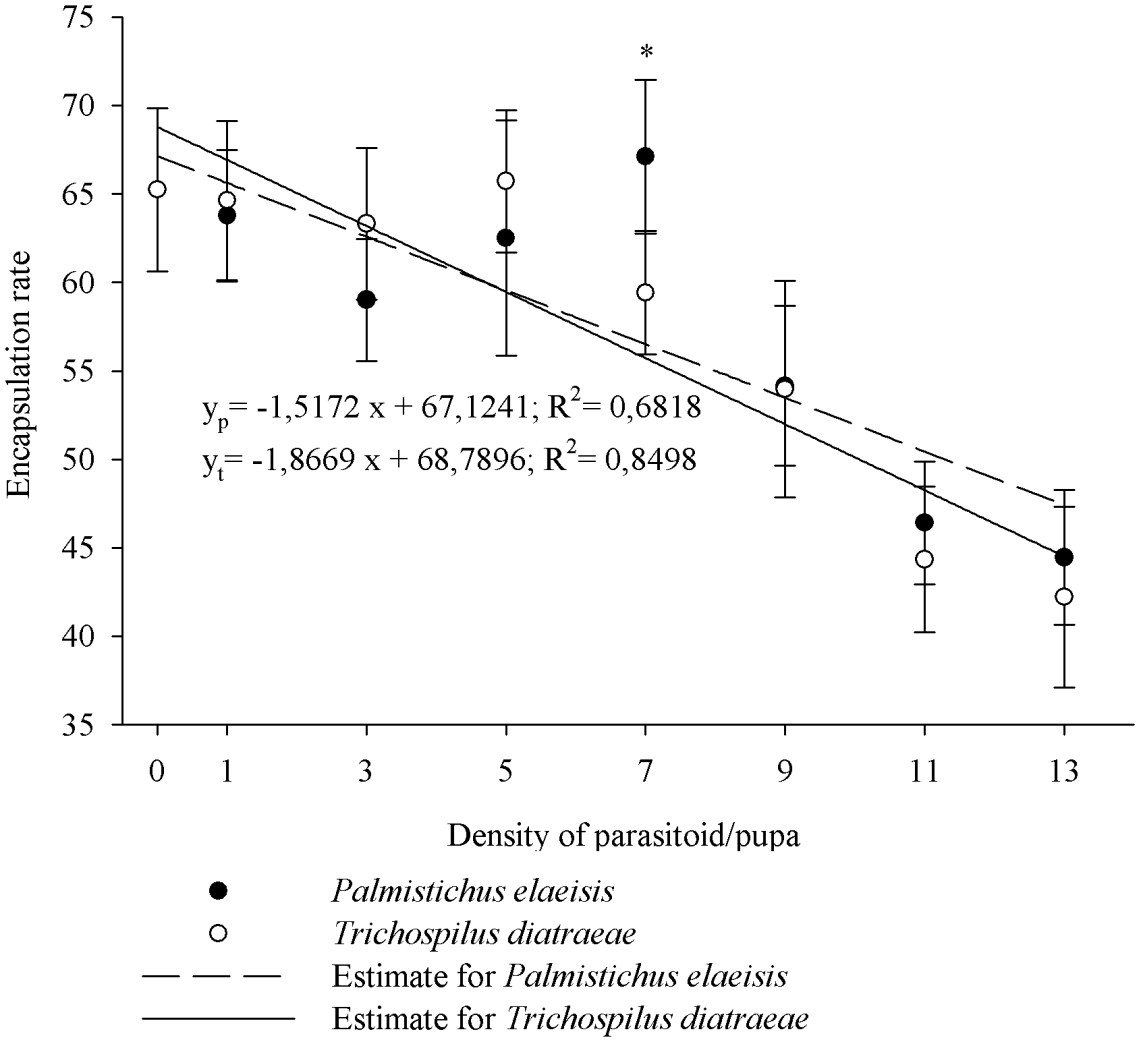
Encapsulation rate pupae of *Anticarsia gemmatalis* pupae parasitized by densities of *Palmistichus elaeisis* or *Trichospilus diatraeae*.

The encapsulation rate was demonstrated by hemocyte adherence to the nylon filaments, followed by darkening of the filaments. Increasing parasitoid density decreased the melanization rate from 65.24±4.61 in non-parasitized pupae to 44.44±3.82 and 42.22±5.10 at higher densities of *P. elaeisis* and *T. diatraeae*, respectively (Figure 5). At 7 parasitoids/pupa, a higher encapsulation rate occurred in pupae parasitized by *P. elaeisis* than by *T. diatraeae* (Figure 5).

## Discussion

Changes in total hemocytes circulating in the haemolymph, and encapsulation rates of parasitized *A.gemmatalis* pupae are critical steps in the suppression of the host defenses by *P. elaeisis* and *T. diatraeae*, allow them to develop immature parasitoids. These features are also likely to be important in the parasitism of pupae of the pest *Acrolepiopsis assectella* (Zeller, 1839) (Lepidoptera: Yponomeutoidae) [15].

The greater reduction in the number of granulocytes in pupae attacked by five or more *P. elaeisis* suggests that host defenses may vary according to the parasitoid species involved. However, since we found similar encapsulation rates for both parasitoids, we suggest that there is link between the number of hemocytes and the humoral response [33], [34].

Changes in the total circulating hemocyte numbers in *A. gemmatalis* pupae parasitized by *P. elaeisis* and *T. diatraeae* are similar to those reported in larvae of *Putella xylostella* L. (Lepidoptera: Plutellidae) parasitized by *Cotesia plutellae* (Kurdjumov) (Hymenoptera: Braconidae) (Ibrahim and Kim, 2006). The polyphagous larval endoparasitoid *Meteorus pulchricornis* (Wesmael, 1835) (Hymenoptera: Braconidae) also induces changes in the numbers of hemocytes of its host [35] indicating that this is a common pathway for host immune suppression in parasitoids. The means by which parasitoids alter the number of circulating hemocytes in the hemolymph has been discussed. Braconidae inject venom associated with teratocytes [35], while Eulophidae inject venom during oviposition [36]. The reduction in the number of circulating hemocytes ensures a favorable environment for the development of the parasitoid larvae and prevents the host producing prophenloxidase, oxygen and nitrogen intermediate reactive species, and melanin [37], [38].

Detailed differences in the suppression of the immune response, and changes in the circulating hemocytes of *A. gemmatalis* by *P. elaeisis* and *T. diatraeae,* suggest that these parasitoids may use different strategies to suppress host immunity, such as through the death of hemocytes or modification of their adhesion properties [1, 30, 39. 40, 41]. These differences may result from toxic substances in ovarian fluids, rich in proteins that induce changes in hemocytes, released by the parasitoids during oviposition [42]. Increases in the concentration of fluids injected during oviposition may enhance the physiological changes in the host hemocytes [43], which may explain the decrease in circulating hemocytes and encapsulation rates in parasitized *A. gemmatalis* pupa.

The similar number of other types of hemocytes circulating in the hemolymph with increasing density of parasitoids may be because these cells play a role in tissue and organ restructuring during the pupal stage, unlike plasmatocytes and granulocytes [44].

The lower melanization of nylon implants in *A. gemmatalis* pupae with the highest densities of parasitoids suggests an effect of parasitoid densities on the population of circulating hemocytes, which participate in the initial process of pathogen recognition and triggering of humoral defenses. Higher densities allow more parasitoid eggs to develop by lowering the ability of the host to encapsulate them [43]. Oviposition of greater numbers of eggs in the same host can counteract a high mortality rate of immature stages of the parasitoid by the host immune system, and result in greater reproductive success of gregarious parasitoids compared to solitary ones [45]. The reduction of humoral and cellular defenses of parasitized *A. gemmatalis* pupae indicates the importance of the gregarious habit for the reproductive success of *P. elaeisis* and *T. diatraeae*. This is demonstrated by the increase in offspring of *Plutella xylostella* (L., 1758) (Lepidoptera: Plutellidae) super-parasitised by *Oomyzus sokolowskii* (Kurdjumov, 1912) (Hymenoptera: Braconidae) [46]. In this case the host immune system is the main factor causing parasitoid mortality in the immature phase, and the degree of encapsulation has strong implications for the reproductive success of its natural enemies [7]–[10], [37]–[47]. Moreover, increase in competition for the food resources provided by the host resulting from super-parasitism may reduce the quality of the parasitoids, and shows the importance of the appropriate density of parasitoids per host individual [48], [49].

Females of the parasitoids *P. elaeisis* and *T. diatraeae* reduce the immune response of the alternative host, *A. gemmatalis*, by reducing the number of circulating hemocytes in the host by increasing the densities of the attacking parasitoids.
